# A metasurface color router facilitating RGB-NIR sensing for an image sensor application

**DOI:** 10.1515/nanoph-2023-0746

**Published:** 2024-01-22

**Authors:** Yoon Jin Hong, Byeong Je Jeon, Yu Geun Ki, Soo Jin Kim

**Affiliations:** Department of Semiconductor Systems Engineering, Korea University, Seoul, Korea; School of Electrical Engineering , Korea University, Seoul, Korea

**Keywords:** spectral routing, metasurface, color filter, CMOS image sensor, nanophotonics

## Abstract

CMOS image sensor (CIS) plays a crucial role in diverse optical applications by facilitating the capture of images in the visible and near-infrared spectra. The enhancement of image resolution in CIS by an increase in pixel density is becoming more significant and realizable with the recent progress of nanofabrication. However, as pixel size decreases towards the diffraction limit, there is an inevitable trade-off between the scale-down of pixel size and the enhancement of optical sensitivity. Recently, to overcome this, an entirely new concept of spectral sensing using a nanophotonic-based color router has been proposed. In this work, we present a metasurface-based spectral router to effectively split the spectrum from visible to near-infrared and redirect through the four optical channels to the targeted pixel surfaces. We optimize the metasurface that simultaneously controls the phases of the transmitted light of targeted spectra, i.e. red (R), green (G), blue (B), and near-infrared (NIR), which is the largest number of channels reported based on a single layered metasurface and has an optical efficiency that surpasses the efficiency of conventional color filter systems.

## 1 Introduction

The development of a CMOS image sensor (CIS) built on a silicon photodetector has been gaining great attention in various fields of IoT applications due to its ability to effectively capture images of visible and near-infrared light. To acquire higher-quality images, it’s important to increase the number of pixels in a given surface area, which enables the increased resolution of images. However, as the pixel size has been reduced to be extremely small, the light receiving area per unit pixel decreases significantly, and this results in decreased optical sensitivity. In particular, as the pixel size approaches the level of the diffraction limit, optical signals can be far below the electrical noise of an integrated CIS system, and such an optical condition ultimately renders a captured image blurry. As such, there remains an inevitable trade-off between the scale-down of pixel sizes and the enhancement of optical sensitivity in effectively detecting optical images.

Meanwhile, the recent progress of metasurfaces has drawn attention from researchers for decades due to the potential ability to control the flow of light for designer purposes. In particular, dielectric metasurfaces constructed from the subwavelength array of lossless nanomaterials have been used to develop practical optical applications, including optical elements and imaging devices. Research on developing lenses [1]–[9], color printing [10], [11], [12], and color filters [13], [14], [15], [16] using nanoscaled structures has been extensively conducted, and metasurface-based devices such as polarimeters [17], [18], [19], [20] and photodetectors [21], [22], [23], [24] and ultrathin imaging elements [25], [26] have been demonstrated. Furthermore, to overcome the aforementioned limit on the loss of light in the CIS system, the concept of a nanophotonic color router has been proposed and intensively studied recently [27]–[35]. Unlike conventional color filters which only transmit targeted spectral bands and reflect or absorb out-of-band power, nanophotonic color routers are designed to re-direct light with a specific targeted spectral band to the designated pixels without seriously deteriorating optical sensitivity. Moreover, nanophotonic routers made from dielectric materials have advantages over conventional filters in that they are less susceptible to environmental conditions such as humidity, temperature, and degradation from light exposure. There have been demonstrations of color routers using metasurfaces that effectively sort the targeted spectrum. Examples of these include the two-channel router, i.e., visible and near-infrared (NIR) [33], [34], or the three-channel router that splits the red (R), green (G), and blue (B) colors to the targeted pixel areas [27]–[31]. A router that affords the separation of four channels, including three visible colors and near-infrared, attracts researchers recently due to its advantages of detecting images at hazed environment and potential applications to IoT sensors for image fusion, face recognition, bio imaging, and many other purposes [36], [37]. However, the development of such router is considered to be more challenging and still elusive due to the increased complexity of phase design and the creation of the library for various meta-structures. Nevertheless, a recent demonstration of an inversely designed nanophotonic router demonstrates efficient splitting of visible and near-infrared light [32], [35].

In this work, we present a metasurface router with four distinct channels constructed from a single nanostructured layer that facilitates the selective separation of the visible and NIR spectra. In contrast to the previously reported three dimensionally structured nanophotonic routers which pose comparatively greater fabrication challenges [32], [35], the proposed design can be fabricated using conventional techniques and effectively separate the spectra into the targeted four bands without significant loss of light occurring in conventional filters. The proposed metasurface-based spectral router shows 32 % averaged optical efficiency over operating RGB and NIR wavelengths, which is the maximum reported number of channels of single layered metasurfaces and exceeds the efficiency of the conventional color filter system.

## 2 Results and discussion

**Figure 1:**
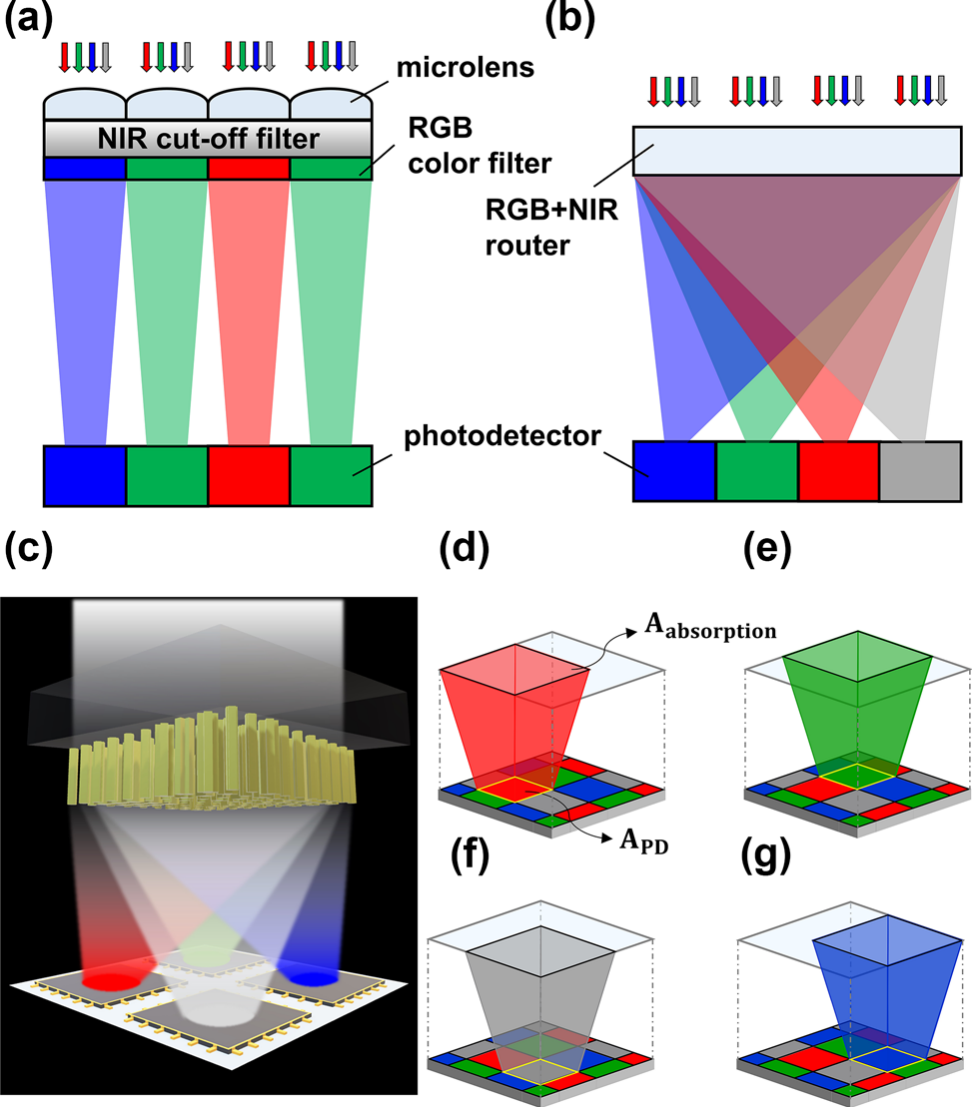
Conceptual schematics of designed spectral router. (a), (b) Conceptual cross-sectional images of conventional color filter system and spectral router system, respectively. (c) Schematic of designed spectral router with different types of meta-atom. (d)–(g) 3D images of the light receiving mechanism for the spectral router. The black boundary line at the top indicates the area where a targeted pixel can collect and absorb the light (*A*_absorption_). The yellow boundary line indicates the physical surface area of a photodetector (*A*_PD_), which implies that *A*_absorption_ is larger than the *A*_PD_.

A comparison between the conventional filter system and the single-layered spectral router is illustrated in Figure 1(a) and (b). In the case of the conventional system (Figure 1(a)), each color filter only transmits the targeted spectrum and reflects or absorbs the remaining parts of spectra to perform selective detection of RGB light. For instance, the “R” cell, which is designed to capture red light, only utilizes a spectrum near the 630 nm wavelength and discards all other wavelengths of light. Based on the working principle, the conventional system with the inclusion of a near infrared channel is restricted to utilizing only 25 % on average of the incoming light.

To overcome such limit and utilize NIR signal effectively, we have designed a single-layered spectral router capable of sorting R, G, B, and NIR light to the four distinct channels (Figure 1(c)). Our design can simultaneously perform selective sorting and focusing without the need for an NIR cut-off filter or microlens elements. For a more comprehensive description, Figure 1(d)–(g) illustrates the light-receiving regions of CIS including the NIR channel. In the case of the red cell of the router design depicted in Figure 1(d), light from adjacent areas (solid black line, *A*_absorption_) is focused onto the targeted photodetector, and a significant portion of received light can be utilized compared to the typical CIS system that only detects the light incident on each designated color filter area. With the working principles, we have developed a single-layer metasurface router for RGB and NIR channels, which could replace the functionality of constituent microlens, color filter, and NIR cut-off filter of conventional system simultaneously.

To optimally perform four channel (R, G, B, and NIR) sorting within a single-layer metasurface, it is important to design the appropriate meta-atoms. The optimization of phase delay, valid and individually controllable for the four channels, is performed simultaneously by adjusting each single meta-atom. Among the previously pioneered available methods [38]–[45], we adopt the property of propagation phase delay of resonant nanostructures which typically support polarization-independent responses. The phase modulation generated by the nanostructure is obtained by Equation (1).

(1) $$\Delta \varphi =\frac{2\pi }{\lambda }\Delta n_{\text{eff}}h$$

**Figure 2:**
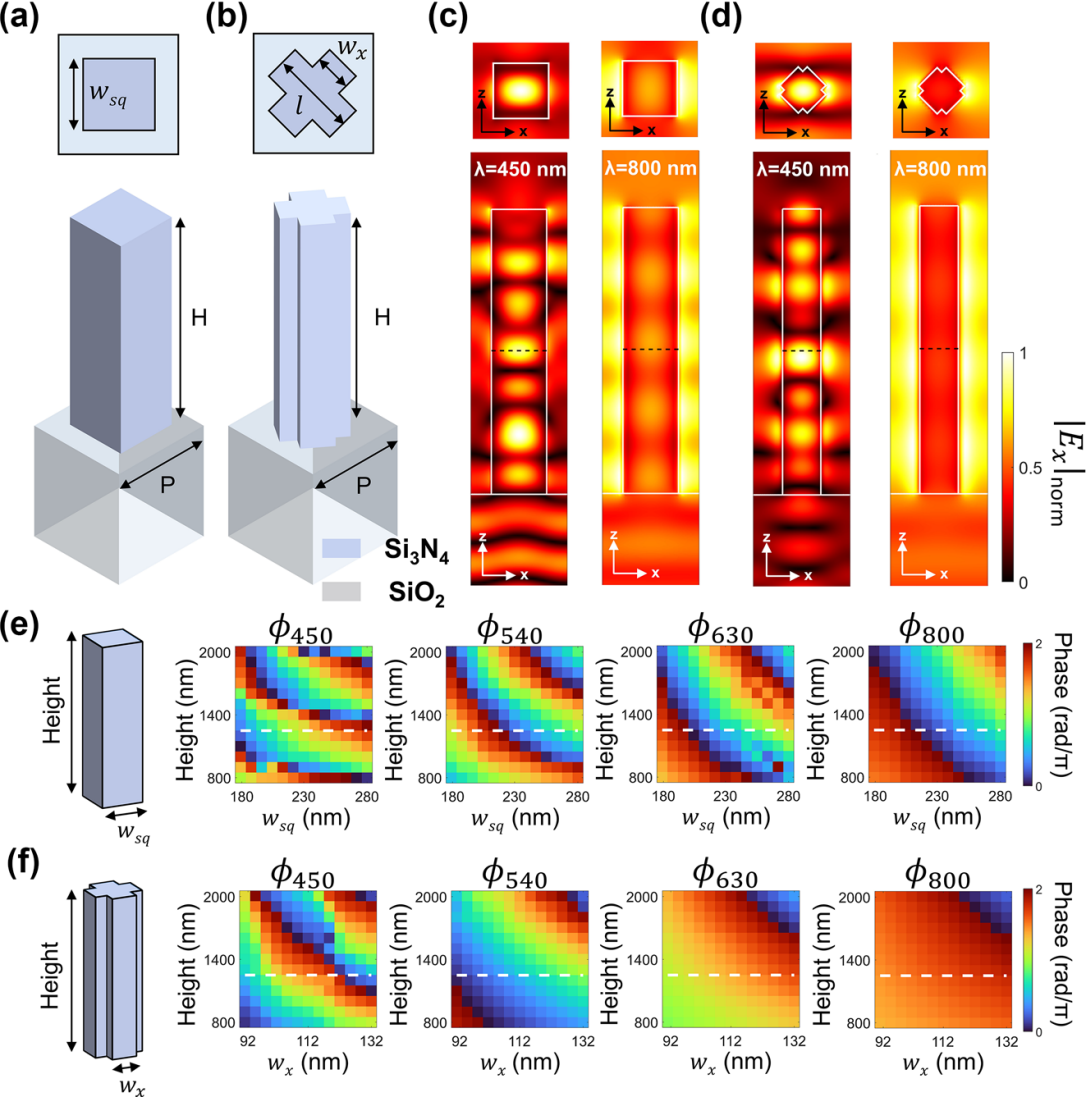
Optimization and analysis of a constituent meta-atom. (a) and (b) Side and top view of a meta-atom. Period (*P*) is fixed to 400 nm, and Si_3_N_4_ based nanostructure is designed on a SiO_2_ substrate. Light is incident to the back side of the substrate and transmitted into free space (*n* = 1). (c) and (d) Normalized electric fields (|**E**_*x*_|_norm_) of a square and cross-shaped nanostructure illustrated in (a) and (b) under the two representative wavelengths of blue (450 nm) and NIR (800 nm), respectively. Width (*w*_*sq*_, *w*_*x*_) and height (*H*) are fixed to be 230 nm, 112 nm (with *l*/*w*_*x*_ = 1.5), and 1250 nm, respectively. The cross-sectional electric fields at the insets are monitored at the height of 630 nm, which are indicated as black dashed lines. (e)–(f) Simulated phase delay of transmitted light by sweeping height and width of the nanostructure under *x*-polarized incident light. Noted that for convenience, *l*/*w*_*x*_ is fixed to 1.5 in (f). White dashed lines indicate the position of *H* = 1250 nm, at which the close to full 2π phase modulation is attained.

Based on the equation, the phase delay (Δ*φ*) depends on the height (*h*) and effective refractive index (Δ*n*_eff_) of the nanostructure. Meta-atoms fabricated using high refractive index materials, such as silicon or germanium, exhibit more effective control of phase delay compared to low-index materials. However, such an advantage comes at the cost of increased optical loss within the visible and near-infrared spectrums. Taking these into consideration, we designed the nanostructure using silicon nitride (Si_3_N_4_), and two different prototype geometries are analyzed as illustrated in Figure 2(a) and (b), which show lossless properties and a relatively larger phase library for design flexibility.

Figure 2(c) and (d) show the electric field images of a meta-atoms with square and cross-sectional shapes, which resonate with different modes at the two representative wavelengths of blue (450 nm) and NIR (800 nm) channels. Incident light is polarized in the *x*-direction and illuminated on the backside of the SiO_2_ substrate.

It is observed in the analysis that the 5th-order Fabry–Pérot (FP) resonance mode is supported at a wavelength of 800 nm (Figure 2(c), right), and the higher-order FP mode with seven anti-nodes at the vertical directions is supported at a shorter wavelength of 450 nm (Figure 2(c), left). The same analysis can be applied to a cross-shaped meta-atom in Figure 2(d), which shows 7th and 4th-order modes at blue and NIR channels. The additional field diagrams at the wavelengths of 540 nm (G) and 630 nm (R) (Supplementary note S1) show different order of modes, which implies that a meta-unit with a height of 1250 nm simultaneously covers low-order and high-order FP modes at the targeted spectral channel.

Figure 2(e) and (f) show the analysis of phase delay as a function of height and width (*w*_*sq*_, *w*_*x*_) at a fixed period of 400 nm. At each targeted wavelength of RGB and NIR, the phases of square-shaped (Figure 2(e)) and cross-shaped (Figure 2(f)) meta-atoms are displayed, respectively. As the height rises, the transmission phase is delayed by more than 2π, which confirms that the designed nanostructures effectively support vertically oriented FP resonances. At the blue channel, a more abrupt change in phase delay occurs due to the higher-order modes of FP resonance compared to the example of the NIR channel where a gradual change in phase modulation occurs by the relatively lower-order of FP resonance. At the height of 1250 nm which is indicated by white dashed lines in the panels, close to full 2π phase modulations are achieved for the four designated channels by altering the width of nanostructures. It is noteworthy that the electric field is more densely concentrated in the squared geometric nanopost (Figure 2(c)) than the field concentration in the cross geometric nanopost (Figure 2(d)), which implies that the square-shaped meta-atom has higher mode index than the cross-shaped one. Such a higher mode index facilitates relatively larger phase variation of squared nanopost (Figure 2(e)) than the phase variation of cross-shaped nanopost (Figure 2(f)) as the width *w*_*sq*_ and *w*_*x*_ equally varies. Thus, the square-shaped meta-atoms primarily address most of phase delay, while the cross-shaped meta-atoms (Figure 2(f)) serve to compensate for the phase delay that the square-shaped meta-atoms cannot sufficiently provide.

**Figure 3:**
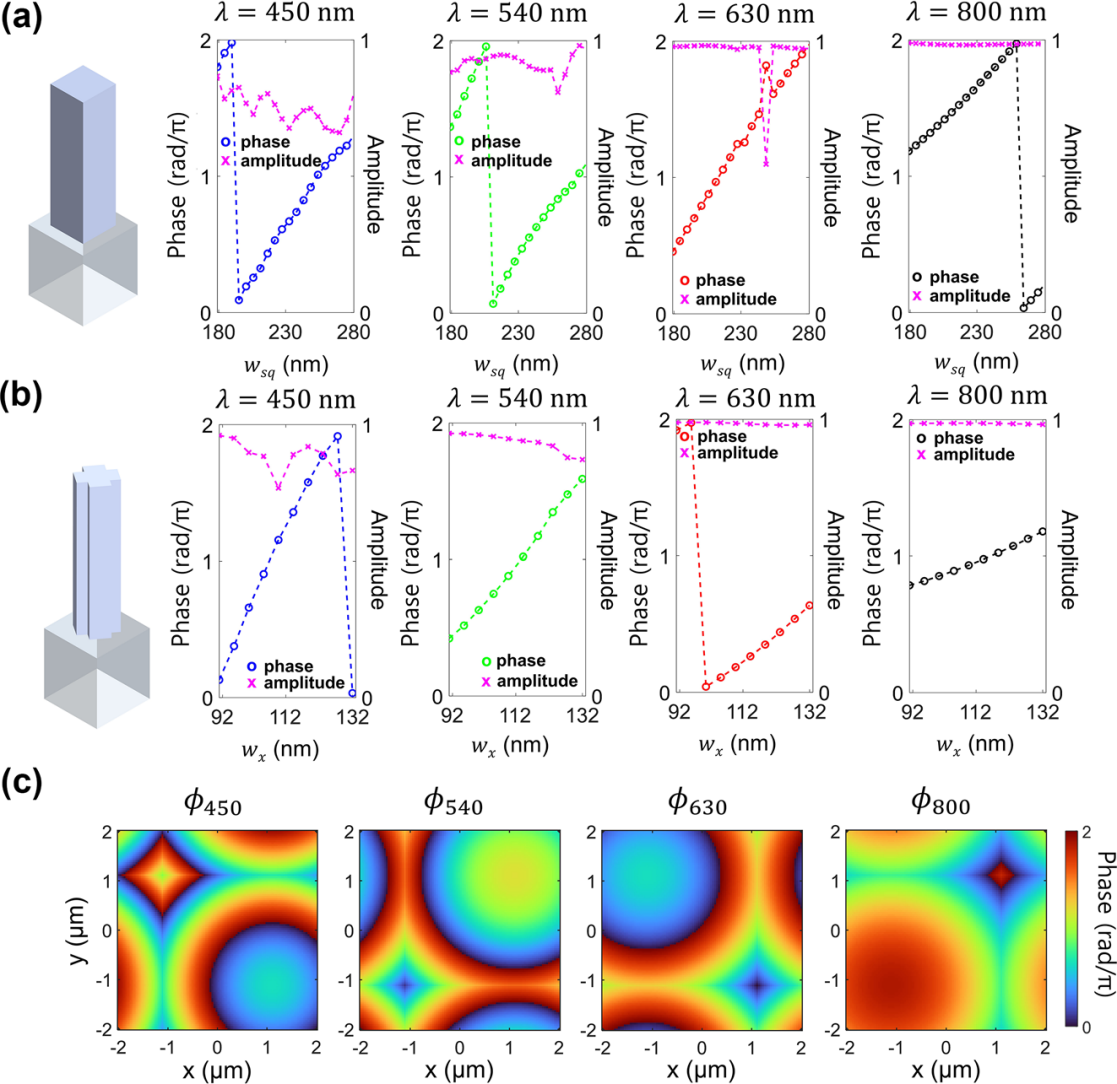
Analysis of phase profiles for the design of metasurface router. (a), (b) Amplitude and phase of transmitted light at square- and cross-shaped meta-atoms. The inset image shows the schematics of meta-atoms. For square-shape, width (*w*_*sq*_) is swept from 180 nm to 280 nm (a), and for cross-shape, width (*w*_*x*_) is swept from 92 nm to 132 nm with the ratio between l and *w*_*x*_ fixed to be 1.5. (c) Designed phase profile for the simultaneous routing of four channels at each center wavelengths of 450 nm, 540 nm, 630 nm, and 800 nm, respectively.

The phase library constructed by the nanostructures with a fixed height of 1250 nm is illustrated in Figure 3(a) and (b). They show the amplitudes and phases of transmitted light from square- and cross-shaped meta-atoms, respectively, with respect to the changes in the cross-sectional size of each nanostructure. As confirmed in Figure 3(a), close to full 2π phase coverage can be obtained in all four channels by adjusting the width of square-shaped meta-atoms (*w*_*sq*_). Despite the effective modulation, at the NIR regime, the square-shaped nanostructure by itself does not provide complete 2π phase modulation due to the effect of lower-order FP mode, which results in relatively smaller phase variations over the control of the geometric structure. To achieve complete control of the 2π phase at the fixed height, the cross-shaped nanostructure is additionally designed and analyzed, which has more degrees of freedom in geometry than the square-shaped nanostructure. By effectively utilizing and allocating the two geometric types of meta-atom on the pixel surface, we obtained high transmission and complete 2π phase coverage for all four spectral channels. Figure 3(b) shows the simulated result with the ratio between *l* and *w*_*x*_ being fixed to 1.5. More detailed phase library is shown in Supplementary note 2.

Since the designed router should operate as a multi-functional lens that simultaneously focuses on four different locations of pixels at each targeted wavelength, it is important to optimize the design which concurrently satisfies the phase distributions at the four spectral channels of R, G, B, and NIR. To satisfy this, we analyze the required phase profile for the positions *x* and *y* at each center wavelength using the following equations.

(2) $$\begin{array}{ll} \phi_{B}\left(x,y\right) & =-\frac{2\pi }{\lambda_{B}}\left(\sqrt{{\left(x-x_{B}\right)}^{2}+{\left(y-y_{B}\right)}^{2}+f^{2}}\right.\\ & \;-\sqrt{{x_{B}}^{2}+{y_{B}}^{2}+f^{2}}+C_{B} \end{array}$$

(3) $$\begin{array}{ll} \phi_{G}\left(x,y\right) & =-\frac{2\pi }{\lambda_{G}}\left(\sqrt{{\left(x-x_{G}\right)}^{2}+{\left(y-y_{G}\right)}^{2}+f^{2}}\right.\\ & \;-\sqrt{{x_{G}}^{2}+{y_{G}}^{2}+f^{2}}+C_{G} \end{array}$$

(4) $$\begin{array}{ll} \phi_{R}\left(x,y\right) & =-\frac{2\pi }{\lambda_{R}}\left(\sqrt{{\left(x-x_{R}\right)}^{2}+{\left(y-y_{R}\right)}^{2}+f^{2}}\right.\\ & \;-\sqrt{{x_{R}}^{2}+{y_{R}}^{2}+f^{2}}+C_{R} \end{array}$$

(5) $$\begin{array}{ll} \phi_{\mathrm{NIR}}\left(x,y\right) & =-\frac{2\pi }{\lambda_{\mathrm{NIR}}}\left(\sqrt{{\left(x-x_{\mathrm{NIR}}\right)}^{2}+{\left(y-y_{\mathrm{NIR}}\right)}^{2}+f^{2}}\right.\\ & \;-\sqrt{{x_{\mathrm{NIR}}}^{2}+{y_{\mathrm{NIR}}}^{2}+f^{2}}+C_{\mathrm{NIR}} \end{array}$$

In each equation, the variables ***x***_***R***, ***G***, ***B***, ***NIR***_ and ***y***_***R***, ***G***, ***B***, ***NIR***_ represent the positions of the focal point for the red, green, blue, and near-infrared channels, respectively, and ***f*** denotes the focal length of the lens. In order to facilitate the effective routing of light to the four channels, each focal point is positioned at the center of four pixel regions on the focal plane. To achieve concurrent modulation of phase throughout all spectral channels and compensate for the errors, the fitting constant ***C*** is employed. The optimal value of the constant ***C*** is achieved by iteratively updating it through cascaded feedback between the temporarily produced phase profile and the accessible phase library of meta-atoms. As illustrated in Figure 3(c), such an iterative process creates a phase distribution profile that closely matches the values of experimentally implementable meta-atoms while minimizing the inaccuracy in the overall control of focusing light at each desired spectrum.

Based on the steps, the values of ***C*** are determined as being *C*_*R*_ = 0, *C*_*G*_ = 0.3307, *C*_*B*_ = 2.9762, and *C*_*NIR*_ = 5.6218, and total 100 points are sampled from the continuous phase profile set to construct the desired metasurface.

**Figure 4:**
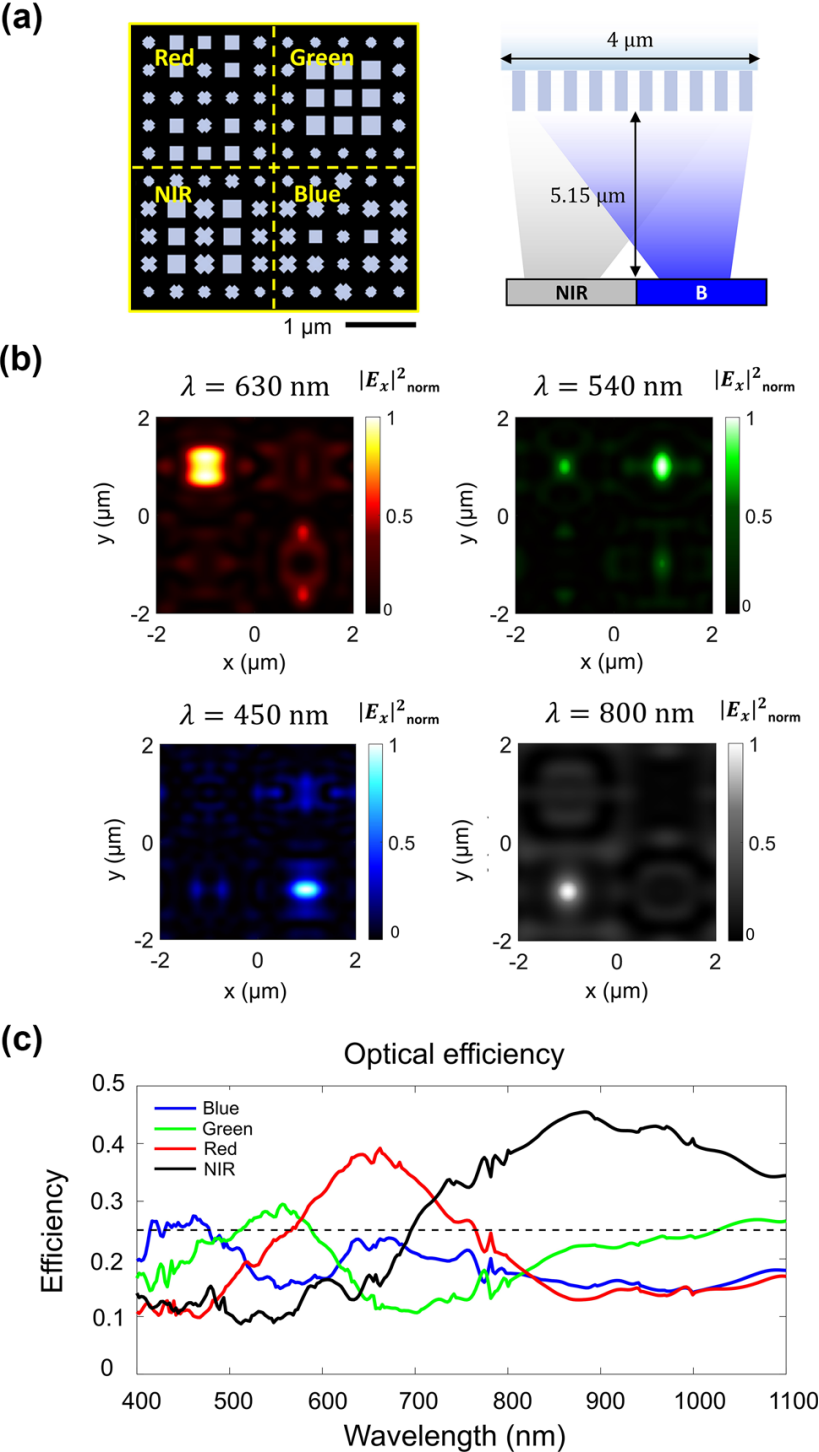
A designed metasurface and its optical performance. (a) Schematic of the overall metasurface-based group pixel which accommodates RGB and NIR. Yellow bold text indicates the location of each pixel (left). The scale bar is 1 μm. Vertical schematic of designed spectral router. The focal length is 5.15 μm (right). (b) Normalized electric field intensities at each center wavelength analyzed under *x*-polarized incident light ( ${\left|E_{x}\right|}^{2}$ _norm_). Focal spots for each wavelength can be observed at each targeted region, which shows the abilities of effective sorting of RGB and NIR. (c) The optical efficiency of the metasurface over a broadband spectral range from visible to NIR. The black dashed line indicates the maximum efficiency that can be achieved in a conventional four channel filter system.

The schematic of the constructed metasurface is illustrated in Figure 4(a). Si_3_N_4_-based meta-atoms are arranged with a combination of nanostructures with different cross-sectional shapes and sizes on a 4 μm × 4 μm metasurface, which is assumed to be periodic. Detailed design information of the metasurface is summarized in Supplementary note 3. To demonstrate the ability of NIR detection, we adopt the conventional RGB Bayer pixel arrangement and replace one green pixel with an NIR pixel. The total transmittance of the metasurface is 64.7 % for blue (*λ* = 450 nm), 76.2 % for green (*λ* = 540 nm), 88.9 % for red (*λ* = 630 nm), and 91.9 % for near-infrared (*λ* = 800 nm), which implies that the designed metasurface is highly transmissive for broadband spectral range. As depicted in Figure 4(b), the electric field intensities at the targeted four channels, i.e. *λ* = 450 nm, 540 nm, 630 nm, and 800 nm, are effectively detected on the focal plane. Although there is a non-negligible spectral crosstalk between the nearby spectrum, the overall power of incident light is predominantly focused near the center of the targeted pixel area, which confirms the effective routing and focusing abilities of the designed metasurface. Such routing phenomena are observed both under the *x*- and *y*-directed polarizations of incident light in a similar fashion (Supplementary note 4). This is due to the geometric properties of the meta-atoms that satisfy C_4_ symmetry and negligible optical coupling with neighboring meta-atoms that mitigate the effect of broken rotational symmetry at the level of the super-cell scale. It is also noteworthy that light is illuminated from SiO_2_ substrate to the metasurface. Such configuration of backside illumination alleviates the efficiency degradation under oblique incident light than the configuration of the direct coupling of light from air. Further optimization can be achieved by placing a metasurface sandwiched between SiO_2_ layers for monolithic integration to CIS.

To evaluate the optical efficiency, we calculate the integrated power density on a targeted 2 μm × 2 μm sub-pixel divided by the total optical power incident on a unit of the metasurface, i.e., 4 μm × 4 μm super-pixel. As exhibited in Figure 4(c), the optical efficiencies over a broad wavelength range of blue (400–500 nm), green (500–600 nm), red (600–700 nm) and NIR (700–1100 nm) are calculated, and the spectral peaks at each pixel exhibit the highest value at the designated spectral range. Specifically, the averaged optical efficiencies for each pixel under the incident light of two orthogonally linear polarizations are 26.5 %, 28.1 %, 37.1 %, and 39.8 % at the aforementioned four wavelengths of blue, green, red, and near-infrared, respectively. It is noteworthy that the averaged efficiencies of the metasurfaces surpass the maximum efficiency of 25 % attainable using conventional color filters of four spectral channels which is outlined as a horizontal dashed line in Figure 4(c). Further reduction in pixel size can be achieved through the compensation of spectral crosstalks and focal lengths (Supplementary note 7). Advanced nanofabrication techniques such as deep reactive ion etching (DRIE) and metal aided chemical etching (MACE) can be used to create the proposed nanostructures with high aspect ratio [46], [47]. We further investigate the effect of fabrication imperfections such as slanted profiles on the performance of the metasurface routers (Supplementary note 5).

**Figure 5:**
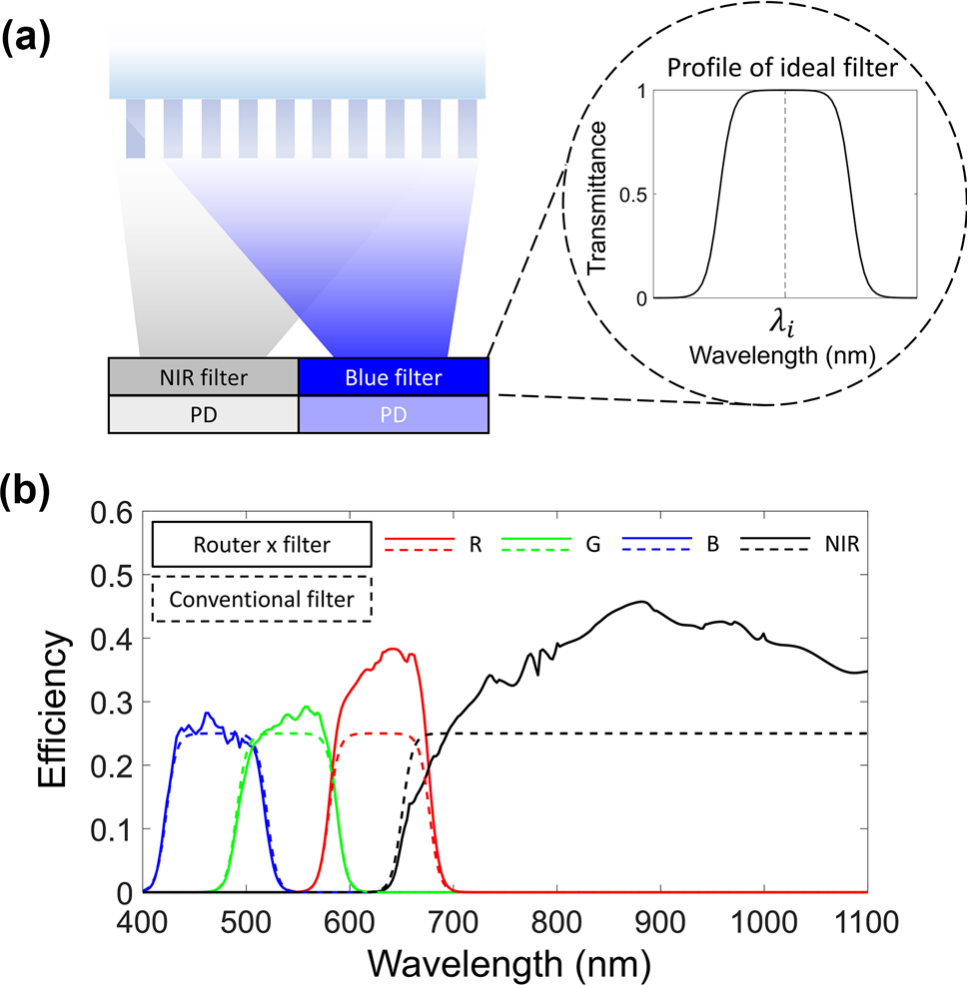
Analysis of hybrid-type metasurface router. (a) Schematical illustration of hybrid-type spectral router. Conventional spectral filter is applied near the focal plane of detecting surfaces. Inset image shows the profile of idealized spectral filter with a peak transmittance 100 % for a single filter. *λ*_*i*_ is 450 nm, 540 nm, 630 nm, and 800 nm for each targeted filter. (b) Optical efficiency of conventional filter system (dashed line) and hybrid spectral router (solid line).

The optical noise caused by the spectral crosstalk can be further minimized by additionally applying the conventional spectral filter above the surface of the photodetector array, which blocks the side band transmission and transmits the routed target spectrum. To realize such a hybrid-type metasurface router, conventional spectral filter layer is integrated with metasurface router as illustrated in Figure 5(a). The spectral shapes for filters are modelled based on the sigmoid function with the variable *k* in the function *σ*(*kx*) being ±0.2 to define the spectral slope of transmission band edge. The profile is configured to exhibit an ideal transmittance of 100 % within the target wavelength range, with a center wavelength denoted by *λ*_*i*_.

The comparison between conventional filter and hybrid-type metasurface router is performed numerically, as depicted in Figure 5(b). Solid line denotes the spectral efficiency of hybrid metasurface router, which exceeds the efficiency of conventional filter array in an overall spectral region as denoted by dashed line. In particular, the efficiency of red and NIR spectra increased approximately by 50 % and 60 %, respectively, compared to the conventional system.

Table 1 shows the summary and comparison of the optical performance with previously pioneered design of nanophotonics or metasurface-based routers. Compared to the previous metasurface, which routes two (visible and NIR) or three (RGB) channels, the main progress of our proposed metasurface is the ability to route four channels, including the near-infrared spectrum. And it achieves an average optical efficiency of over 30 % for the broadband wavelength regime, which still exceeds the efficiency limit of conventional color filter systems.

**Table 1:** Comparison with previously pioneered routers.

Ref	Material	Configuration	Operating wavelength (nm)	Efficiency (%)
[48]	TiO_2_	Single cubic	400 nm, 500 nm	Not reported
[49]	Ag	Single nanorod	^a^Not specifically mentioned	Not reported
[27]	SiN	Single-layered metasurface	400–700 nm	25 % (B), 50 % (G), 65 % (R)
[29]	GaN	Single-layered metasurface	430 nm, 532 nm, 633 nm	^b^15.9 % (B), 37.9 % (G), 27.6 % (G), 38.3 % (R)
[31]	Si_3_N_4_	Single-layered metasurface	400–700 nm	49 % (B), 59 % (G), 58 % (R)
[32]	Si_3_N_4_, TiO_2_	3D metamaterial (inverse design)	400–800 nm	^c^>99 % (B, G, R, NIR)
[33]	SiN	Single-layered metasurface	400–1100 nm	^d^59 % (Vis), 60 % (NIR)
[34]	Si_3_N_4_	Single-layered metasurface	400–1000 nm	^e^71.2 % (Vis), 80.36 % (NIR)
[35]	Si_3_N_4_	3D metamaterial (inverse design)	400–800 nm	^f^29 % (B), 38 % (G), 39 % (R), 36 % (NIR)
This work	Si_3_N_4_	Single-layered metasurface	400–1100 nm	26.5 % (B), 28.6 % (G), 37.1 % (R), 39.1 % (NIR)

All described efficiencies (except [33]) are calculated (or measured) on a center wavelength. For [27], [29], [31], two pixels for green channel are used. ^a^Operating channels are defined as red-blue-red or green-red-green for a single Ag nanorod. ^b^Efficiencies are measured by the single wavelength laser illuminated on the spectral router. The second and third efficiencies are for the green pixels. ^c^Over 99 % of optical efficiency has been achieved in this report. However, the proposed design is based on the inverse design multi-layered metasurface. ^d^Two channels (visible and NIR) were adopted. Described efficiencies are averaged over the operating wavelength range. ^e^Measured efficiencies at the center wavelengths are described above. ^f^Similar with [32], the inverse design multi-layered metasurface is provided in the research.

## 3 Conclusions

To summarize, we present a single layered metasurface capable of sorting a broad range of light to four spectral channels that the silicon photodetector can respond to. The basic optical properties of a meta-atom are additionally analyzed, which support FP resonance and afford full coverage of phase delays. We believe that the proposed design offers the potential solutions to realize selective sorting of light over a broad spectral range for the applications of miniaturized pixel-based image sensors. As a prospective research direction, further scaling down of the metasurface router for reduced pixels should be investigated, as should the development of advanced fabrication techniques and the exploration of various candidates of emerging materials in order to realize advanced nanophotonic color routers for miniaturization, simplification, and enhanced quality of imaging for future optical devices.

## 4 Methods

### 4.1 Optical simulation

To optimize and evaluate the optical performance of the device, commercial finite-difference time-domain (FDTD) software from Lumerical Solutions is used. Arrays of Si_3_N_4_ nanostructure with a 1250 nm height (refractive index obtained from the data measured by Phillip in 1973) are arranged on the SiO_2_ substrate (refractive index from the Handbook of Optical Constants of Solids by Edward D. Palik). The period of the unit cell is designed to be 400 nm, and the metasurface array is designed as 4 μm by 4 μm for *x*- and *y*-directions. Periodic boundary conditions are used in the *x*- and *y*-directions, and perfectly matched layer (PML) boundary conditions are applied along the *z*-direction for the simulation of both a unit cell and a metasurface. Transmittance and optical efficiencies are calculated by monitoring transmitted power at the focal plane.

## Supplementary Material

Supplementary Material Details
