# Supplementary material for: A metasurface color router facilitating RGB-NIR sensing for an image sensor application
Source: Nanophotonics. 2024 Jan 22;13(8):1407–15. doi: 10.1515/nanoph-2023-0746 (PMC11635924; doi:10.1515/nanoph-2023-0746)
Supplement: Supplementary file 1 — Supplementary Material Details [file j_nanoph-2023-0746_suppl_001.docx]

Supplementary Note

**A metasurface color router facilitating RGB-NIR sensing for an image sensor application**

Yoon Jin Hong^1^, Byeong Je Jeon^2^, Yu Geun Ki^2^, and Soo Jin Kim^2,*^

*^1^Department of Semiconductor Systems Engineering, Korea University, Seoul, Korea*

*^2^School of Electrical Engineering, Korea University, Seoul, Korea*

E-mail: kimsjku@korea.ac.kr

**Supplementary note 1.** Analysis of a resonant meta-atom

**

**

**Figure S1.** Normalized $\left| \mathbf{E}_{x} \right|$ field of a resonant meta-atom at the targeted wavelengths of R (630 nm), G (540 nm), B (450 nm) and NIR (800 nm). The width of square-shaped meta-atom is 230 nm ($w_{sq}$) and $w_{x}$=112 nm, $l/w_{x}$=1.5 for cross-shaped meta-atom. (a), (b) Different orders of Fabry-Perót resonance modes are supported at each targeted wavelength under square and cross-shaped meta-atom. Cross-sectional electric field at XY plane is illustrated at each vertical position indicated as black dashed lines in the field profiles of XZ plane.

**Supplementary note 2.** Phase library of cross-shaped meta-atom


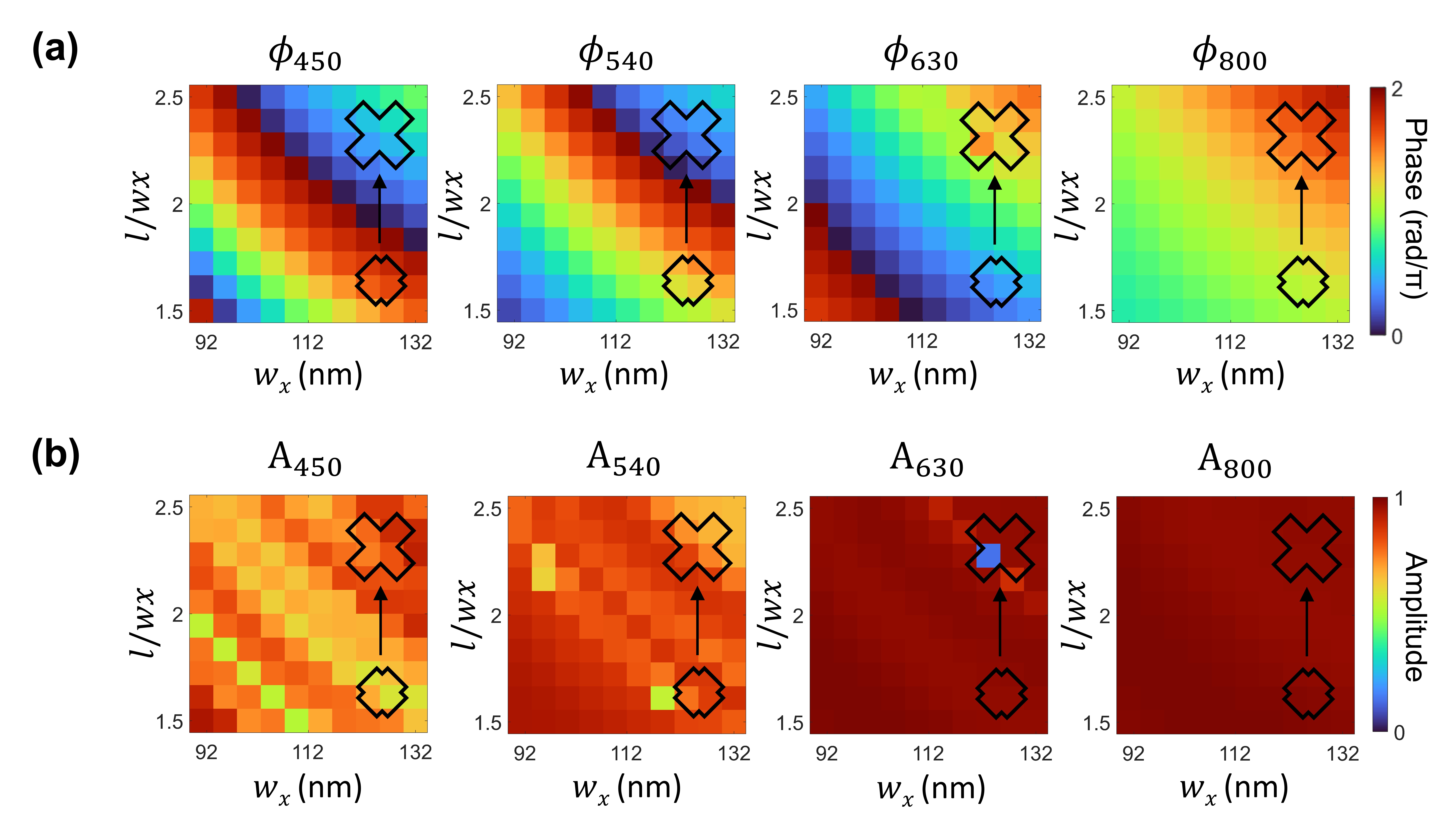


**Figure S2.** Phase library of cross-shaped meta-atom. (a) Phase variation observed by adjusting the value of $w_{x}$ (92 nm – 132 nm) and $l/w_{x}$ (1.5 – 2.5). (b) Transmission amplitude simulated under same condition.

**Supplementary note 3.** Parameters of meta-atoms used in the spectral router design

**Table S1.** Parameters of square-shaped meta-atom in metasurface

| No. | S1 | S2 | S3 | S4 | S5 | S6 | S7 | S8 | S9 |
| --- | --- | --- | --- | --- | --- | --- | --- | --- | --- |
| *w_sq_* (nm) | 192 | 193 | 203 | 206 | 246 | 260 | 261 | 262 | 274 |

** Values are rounded to the second decimal place if necessary.

**Table S2.** Parameters of cross-shaped meta-atom in metasurface

| No. | X1 | X2 | X3 | X4 | X5 | X6 | X7 | X8 |
| --- | --- | --- | --- | --- | --- | --- | --- | --- |
| *l* (nm) | 138 | 168.67 | 144.67 | 176.81 | 198.25 | 241.11 | 173.75 | 241.01 |
| *w_x_* (nm) | 92 | 92 | 96.44 | 96.44 | 96.44 | 96.44 | 100.89 | 100.89 |
| No. | X9 | X10 | X11 | X12 | X13 | X14 | X15 | X16 |
| *l* (nm) | 169.70 | 263.33 | 164.67 | 201.26 | 285.56 | 239.38 | 233.85 | 286 |
| *w_x_* (nm) | 105.33 | 105.33 | 109.78 | 109.78 | 114.22 | 123.11 | 127.56 | 132 |

** Values are rounded to the second decimal place if necessary.

**Table S3.** Meta-atom location in metasurface design

| **X9** | **S3** | **S1** | **S3** | **X9** | **X1** | **X1** | **X1** | **X1** | **X1** |
| --- | --- | --- | --- | --- | --- | --- | --- | --- | --- |
| **X2** | **S4** | **X12** | **S4** | **X2** | **X11** | **S9** | **S8** | **S9** | **X11** |
| **X2** | **X7** | **X5** | **X7** | **X2** | **X2** | **S7** | **S5** | **S7** | **X2** |
| **X2** | **S4** | **X12** | **S4** | **X2** | **X11** | **S9** | **S8** | **S9** | **X11** |
| **X9** | **S3** | **S1** | **S3** | **X9** | **X1** | **X1** | **X1** | **X1** | **X1** |
| **X1** | **X5** | **X2** | **X5** | **X1** | **X3** | **X3** | **X15** | **X3** | **X3** |
| **X6** | **S6** | **X16** | **S6** | **X6** | **X10** | **X14** | **X2** | **X14** | **X10** |
| **X6** | **S5** | **X13** | **S5** | **X6** | **X8** | **S2** | **X4** | **S2** | **X8** |
| **X6** | **S6** | **X16** | **S6** | **X6** | **X10** | **X14** | **X2** | **X14** | **X10** |
| **X1** | **X5** | **X2** | **X5** | **X1** | **X3** | **X3** | **X15** | **X3** | **X3** |

**Supplementary note 4.** Analysis of the polarization response of the metasurface





**Figure S3.** Optical properties of the metasurfaces under y-directed polarization. (a) Electric field intensity under the incident light with y-directed polarization, which indicates the similar focusing abilities of the metasurface under x- directional polarization described in Fig.4(b). (b), (c) Optical efficiency of the metasurface under x-directed polarization (b) and y-directed polarization (c). Both results show similar spectral distribution, indicating the polarization insensitivity of the designed metasurface.

**Supplementary note 5.** Tolerance for the fabrication errors

Given the intricate structure of the router, which consists of meta-atoms with fine geometry, several potential fabrication issues are investigated, including a curved edge of the meta-atom (Figure S4), variations in size (Figure S5), and the effect of tilted etching on the optical performance (as illustrated in Figure S6). Firstly, we begin by determining the degree of tolerance by manipulating the finely sharped geometry into a curved smooth edge as shown in Figure S4. The overall spectra exhibit a consistent trend, for the variations in edge geometry. While the plasmonic resonances are highly dependent on sharped corners due to the collective oscillations of free electrons, dielectric resonances are relatively resistant to such variations in corner edges due to the oscillations of bound electrons. Furthermore, we analyze the variation of nanostructure size (Fig S5) and the trapezoidal vertical profiles occurred in etching processes (Fig S6). Except for the performance of the blue pixel, the figures show that the targeted pixels are relatively resistant to such fabrication imperfections.


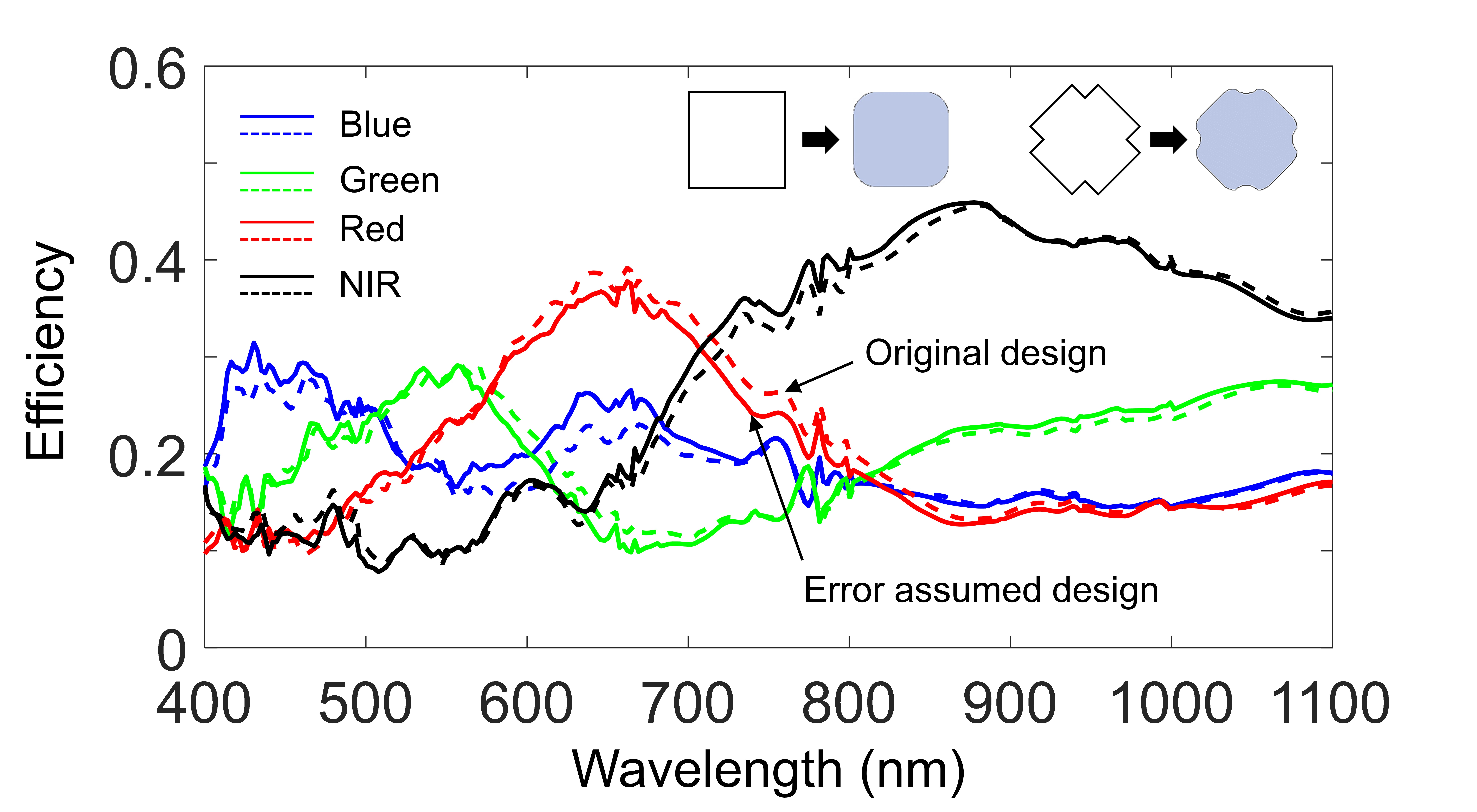


**Figure S4.** Effect of the curvature of the meta-atom's edge. The solid line represents the spectral efficiency of the original design, and the dotted line represents the efficiency estimated under curved-edge conditions. The provided graphic depicts the proposed design of the meta-atom for the simulation.





**Figure S5.** Effect of the variations of width on the sorting efficiency. 𝜶 indicates the size variations of the constituent meta-atoms.





**Figure S6.** Effect of slanted side of the structure on the sorting efficiency. Inset image shows trapezoidal cross-section of the meta-atom, which assumes the tilted profile situation. 𝜷 indicates the difference between top and bottom width.

**Supplementary note 6.** Pixel scaling down

To demonstrate the possibility of smaller pixel design, scaling down of the metasurface router has been designed and optimized with pixel size of 1.6 μm based on the phase library already constructed from the original design. The pixel size decreases by 20%, and the focal length increases to 5.75 μm, which is 11.7% increase compared to the original router design. This analysis demonstrates the possible realization of RGB-NIR router with reduced pixel size. Additional research on the reduction of router size is required with the design incorporating unit-cell re-optimization, such as a decrease in unit-cell period and further analysis of various shapes of nanostructure candidates.





**Figure S7.** Optical performance of RGB-NIR router with reduced pixel size of 1.6 μm. (a) Electric field distribution at the focal plane of the reduced pixel. (b) Optical sorting efficiency over operating spectra including visible and NIR (400 – 1100 nm). Inset image shows the schematic of router design, and each colored solid line indicates the simulated efficiency at each targeted pixel.

| No. | S1 | S2 |
| --- | --- | --- |
| *w_sq_* (nm) | 279 | 280 |

**Table S4.** Parameters of square-shaped meta-atom in metasurface

** Values are rounded to the second decimal place if necessary.

**Table S5.** Parameters of cross-shaped meta-atom in metasurface

| No. | X1 | X2 | X3 | X4 | X5 | X6 | X7 | X8 |
| --- | --- | --- | --- | --- | --- | --- | --- | --- |
| *l* (nm) | 138 | 168.67 | 178.89 | 189.11 | 144.67 | 187.53 | 162.54 | 173.75 |
| *w_x_* (nm) | 92 | 92 | 92 | 92 | 96.44 | 96.44 | 100.89 | 100.89 |
| No. | X9 | X10 | X11 |  |  |  |  |  |
| *l* (nm) | 184.96 | 196.17 | 178 |  |  |  |  |  |
| *w_x_* (nm) | 100.89 | 100.89 | 118.67 |  |  |  |  |  |

** Values are rounded to the second decimal place if necessary.

**Table S6.** Meta-atom location in metasurface design

| **X9** | **X3** | **X3** | **X9** | **X10** | **S1** | **S1** | **X10** |
| --- | --- | --- | --- | --- | --- | --- | --- |
| **X4** | **X2** | **X2** | **X4** | **X6** | **S2** | **S2** | **X6** |
| **X4** | **X2** | **X2** | **X4** | **X6** | **S2** | **S2** | **X6** |
| **X9** | **X3** | **X3** | **X9** | **X10** | **S1** | **S1** | **X10** |
| **X11** | **X8** | **X8** | **X11** | **X11** | **X1** | **X1** | **X11** |
| **X1** | **X7** | **X7** | **X1** | **X1** | **X5** | **X5** | **X1** |
| **X1** | **X7** | **X7** | **X1** | **X1** | **X5** | **X5** | **X1** |
| **X11** | **X8** | **X8** | **X11** | **X11** | **X1** | **X1** | **X11** |
